# *Wolbachia* Enhances West Nile Virus (WNV) Infection in the Mosquito *Culex tarsalis*

**DOI:** 10.1371/journal.pntd.0002965

**Published:** 2014-07-10

**Authors:** Brittany L. Dodson, Grant L. Hughes, Oluwatobi Paul, Amy C. Matacchiero, Laura D. Kramer, Jason L. Rasgon

**Affiliations:** 1 Department of Entomology, Pennsylvania State University, University Park, Pennsylvania, United States of America; 2 Center for Infectious Disease Dynamics, Pennsylvania State University, University Park, Pennsylvania, United States of America; 3 Huck Institutes of the Life Sciences, Pennsylvania State University, University Park, Pennsylvania, United States of America; 4 Department of Biological Sciences, University of Maryland Baltimore County, Baltimore, Maryland, United States of America; 5 Arbovirus Laboratories, Wadsworth Center, New York State Department of Health, Slingerlands, New York, United States of America; 6 School of Public Health, State University of New York at Albany, Albany, New York, United States of America; Mahidol University, Thailand

## Abstract

Novel strategies are required to control mosquitoes and the pathogens they transmit. One attractive approach involves maternally inherited endosymbiotic *Wolbachia* bacteria. After artificial infection with *Wolbachia*, many mosquitoes become refractory to infection and transmission of diverse pathogens. We evaluated the effects of *Wolbachia* (*w*AlbB strain) on infection, dissemination and transmission of West Nile virus (WNV) in the naturally uninfected mosquito *Culex tarsalis*, which is an important WNV vector in North America. After inoculation into adult female mosquitoes, *Wolbachia* reached high titers and disseminated widely to numerous tissues including the head, thoracic flight muscles, fat body and ovarian follicles. Contrary to other systems, *Wolbachia* did not inhibit WNV in this mosquito. Rather, WNV infection rate was significantly higher in *Wolbachia*-infected mosquitoes compared to controls. Quantitative PCR of selected innate immune genes indicated that REL1 (the activator of the antiviral Toll immune pathway) was down regulated in *Wolbachia*-infected relative to control mosquitoes. This is the first observation of *Wolbachia*-induced enhancement of a human pathogen in mosquitoes, suggesting that caution should be applied before releasing *Wolbachia*-infected insects as part of a vector-borne disease control program.

## Author Summary

Current methods to control mosquitoes and the pathogens they transmit are ineffective, partly due to insecticide and drug resistance. One novel control method involves exploiting naturally occurring *Wolbachia* bacteria in insects. *Wolbachia* are bacterial symbionts that are attractive candidates for mosquito-borne disease control due to their ability to inhibit pathogens infecting humans. Additionally, *Wolbachia* affects insect reproduction to facilitate its own transmission to offspring, which has been exploited to establish the bacterium in naturally uninfected field populations. Most *Wolbachia* pathogen control research has focused on *Aedes* and *Anopheles* mosquitoes, but *Culex* mosquitoes also transmit pathogens that affect human health. We evaluated impacts of *Wolbachia* infection on West Nile virus (WNV) in the naturally uninfected mosquito *Culex tarsalis*. *Wolbachia* was able to efficiently establish infection in *Cx. tarsalis* but contrary to other studies, *Wolbachia* enhanced rather than inhibited WNV infection. Enhancement occurred in conjunction with suppression of mosquito anti-viral immune gene expression. This study indicates that *Wolbachia* control strategies to disrupt WNV via pathogen interference may not be feasible in *Cx. tarsalis*, and that caution should be used when releasing *Wolbachia* infected mosquitoes to control human vector-borne diseases.

## Introduction

Efforts to control vector-borne pathogens have been hindered by evolution of insecticide resistance and failing drug therapies. Evidence suggests bed nets and indoor residual spraying with insecticides are losing efficacy in developing countries [1], [2]. To improve the sustainability and efficacy of control efforts, alternative vector control strategies are being considered, including methods that suppress the pathogen instead of the vector [3], [4]. *Wolbachia* are a genus of maternally-inherited bacterial endosymbionts that naturally occur in numerous arthropod taxa [5]. *Wolbachia* can inhibit viruses and parasites in fruit flies and mosquitoes [6]–[11] and influence reproduction of their host to facilitate spread through populations [12]. Mosquito-borne disease management programs that use *Wolbachia* are currently under investigation [13]. In field trials in Australia, *Wolbachia* reached fixation in naturally uninfected populations of *Aedes aegypti* [11] and the DENV blocking phenotype has been maintained [14], but the impacts of *Wolbachia* on reducing the incidence of disease are yet to be investigated.

Pathogen interference conferred by *Wolbachia* depends on various factors, including *Wolbachia* strain, pathogen type, infection type (natural versus artificial) and host and is not a guarantee [7], [15], [16]. For example, *Wolbachia* increases *Plasmodium berghei, P. yoelii and P. gallinaceum* oocyst loads in *Anopheles gambiae*, *An. stephensi*, and *Aedes fluviatilis*, respectively [17]–[19], and *P. relictum* sporozoite prevalence in *Culex pipiens* [20]. These *Wolbachia*-mediated pathogen enhancement studies suggest that careful examination of *Wolbachia* is required, since the bacterium influences insect-pathogen interactions in ways that may negatively impact pathogen control efforts.

Few studies have investigated the effect of *Wolbachia* on pathogen transmission by *Culex* mosquitoes, despite the fact they transmit viruses impacting human health [9], [21], [22]. *Culex tarsalis* is a mosquito species associated with agriculture and urban areas in the western United States [23] and is highly competent for West Nile virus (WNV), St. Louis encephalitis virus (SLEV) and western equine encephalitis virus (WEEV) [24]–[26]. *Cx. tarsalis* are naturally uninfected with *Wolbachia* [27]. We established *Wolbachia* infections in this mosquito by intrathoracic injection of purified symbionts into adult females, characterized the extent of the infection by fluorescence *in situ* hybridization and quantitative PCR, and assessed the ability for *Wolbachia* to block WNV infection, dissemination and transmission at multiple time points. We found that, in contrast to other systems, *Wolbachia* infection enhanced WNV infection rates 7 days post-blood feeding. This is the first observation of *Wolbachia*-induced enhancement of a human pathogen in mosquitoes and suggests that caution should be applied before using *Wolbachia* as part of a vector-borne disease control program.

## Methods

### Ethics statement

Mosquitoes were maintained on commercially available bovine blood using a membrane feeder. WNV infection experiments were performed under biosafety-level 3 (BSL3) and arthropod-containment level 3 (ACL3) conditions.

### Mosquitoes, *Wolbachia*, and West Nile virus

The *Cx. tarsalis* YOLO strain was used for experiments. The colony was originally established from Yolo County, CA in 2009. Mosquitoes were reared and maintained at 27°C±1°C, 16∶8 hour light∶dark diurnal cycle at approximately 45% relative humidity in 30×30×30 cm cages. The *w*AlbB *Wolbachia* strain was purified from *An. gambiae* Sua5B cells according to published protocols [28]. Viability and density of the bacteria was assessed using the Live/Dead BacLight Kit (Invitrogen) and a hemocytometer. The experiment was replicated twice; *w*AlbB concentrations were: replicate one, 5.3×10^9^ bacteria/mL; replicate two, 1.3×10^11^ bacteria/mL. Two- to four-day-old adult female *Cx. tarsalis* were anesthetized with CO_2_ and intrathoracically (IT) injected with approximately 0.1 uL of either *w*AlbB or Schneider's insect media (Sigma Aldrich) as a control. Mosquitoes were provided with 10% sucrose *ad libitum* and maintained at 27°C in a growth chamber. WNV strain WN02-1956 (GenBank: AY590222) was originally isolated in African green monkey kidney (Vero) cells from an infected American crow in New York in 2003 [29] and amplified in *Aedes albopictus* cells (C6/36) to a final titer of 5.0×10^9^ PFU/ml. WNV was added to 5 mL defibrinated bovine blood (Hema-Resource & Supply, Aurora, OR) with 2.5% sucrose solution. Replicate titers were: replicate one, 8.0×10^7^ PFU/mL; replicate two, 3.0×10^7^ PFU/mL. Seven days post *Wolbachia* injection mosquitoes were fed a WNV infectious blood meal via Hemotek membrane feeding system (Discovery Workshops, Accrington, UK) for approximately one hour. Partially- or non-blood fed females were excluded from the analysis.

### Fluorescence *in situ* hybridization (FISH) and microscopy

To characterize *Wolbachia* infections in *Cx. tarsalis* tissues, we performed fluorescence *in situ* hybridization (FISH) on mosquitoes at 12 dpi according to published protocols [10] with slight modifications. Briefly, mosquitoes were fixed in acetone, embedded in paraffin wax and sectioned with a microtome. Slides were dewaxed with three successive xylene washes for 5 minutes, followed by two 5-minute washes with 100% ethanol and one wash in 95% ethanol before treatment with alcoholic hydrogen peroxide (6% H_2_O_2_ in 80% ethanol) for 3 days to minimize autofluorescence. Sectioned tissues were hybridized overnight in 1 ml of hybridization buffer (50% formamide, 5× SSC, 200 g/liter dextran sulfate, 250 mg/liter poly(A), 250 mg/liter salmon sperm DNA, 250 mg/liter tRNA, 0.1 M dithiothreitol [DTT], 0.5× Denhardt's solution) with *Wolbachia* specific probes W1 and W2 labeled with a 5-prime rhodamine fluorophore [30]. After hybridization, tissues were successively washed three times in 1× SSC, 10 mM DTT and three times in 0.5× SSC, 10 mM DTT. Slides were mounted with SlowFade Gold antifade reagent (Invitrogen) and counterstained with DAPI (Roche). Images were captured with a LSM 510 META confocal microscope (Zeiss) and epifluorescent BX40 microscope (Olympus). Images were processed using LSM image browsers (Zeiss) and Photoshop 7.0 (Adobe) software. No-probe, competition probe and RNAse treatment controls were conducted (Figure S1).

### Vector competence for WNV

Virus infection and transmission assays were performed as described at 7 and 14 days post blood feeding [31]–[33]. Female mosquitoes were anesthetized with triethylamine (Sigma, St. Louis, MO), legs from each mosquito were removed and placed separately in 1 mL mosquito diluent (MD: 20% heat-inactivated fetal bovine serum [FBS] in Dulbecco's phosphate-buffered saline, 50 ug/mL penicillin/streptomycin, 50 ug/mL gentamicin and 2.5 ug/mL fungizone). The proboscis of each mosquito was positioned in a tapered capillary tube containing 10 uL of a 1∶1 solution of 50% sucrose and FBS to induce salivation. After 30 minutes, the contents were expelled into 0.3 mL MD and bodies were placed individually into 1 mL MD. Mosquito body, legs and salivary secretion samples were stored at −70°C until tested for WNV presence and *Wolbachia* titers. Mosquito bodies and legs were homogenized for 30 seconds utilizing Qiagen Tissue Lyser at 24 cycles/second, followed by clarification via centrifugation for one minute. Mosquito samples were tested for WNV infectious particles by plaque assay on Vero cells [34]. Infection was defined as the proportion of mosquitoes with WNV positive bodies. Dissemination and transmission were defined as the proportion of infected mosquitoes with WNV positive legs and salivary secretions, respectively. Proportions were compared using Fisher's exact test. The experiment was replicated twice.

### Quantitative real-time PCR (qPCR) of *Wolbachia* density

To evaluate *Wolbachia* density in individual mosquitoes from vector competence experiments, DNA was extracted using DNeasy Blood and Tissue kits (Qiagen) and used as template for qPCR on a Rotor Gene Q (Qiagen) with the SYBR green PCR kit (Qiagen). *Wolbachia* DNA was amplified with primers Alb-GF and Alb-GR [35] and was normalized to the *Cx. tarsalis* actin gene [36] (Table 1). *Wolbachia* to host genome ratios were calculated using Qgene [37]. PCRs were performed in duplicate. Comparisons of *Wolbachia* titers between treatments were analyzed using Mann-Whitney U test.

**Table 1:** Primers used for qPCR.

Primer	Sequence 5′-3′	Reference
REL1-F	GCGACTTTGGCATCAAGCTC	This study
REL1-R	GTTCGACCGGAGCGTAGTAG	
REL2-F	GTCGAGATGGCCAAAACGATG	This study
REL2-R	ACTCACTCATATTGTTGATGGCATT	
CACTUS-F	GACCTGTGCAAGAGTCTGCT	This study
CACTUS-R	ACGTATCACCATCGTCGTTC	
DEFENSIN-F	TTGTTTGCTTCGTTGCTCTTT	This study
DEFENSIN-R	ATCTCCTACACCGAACCCACT	
DIPTERICIN-F	CCCAGCGCTGCTTACTT	This study
DIPTERICIN-R	CATCATCCAGGCCGAGAAC	
ALB-GF	GGTTTTGCTTATCAAGCAAAAG	[35]
ALB-GR	GCGCTGTAAAGAACGTTGATC	
ACTIN-F	GACTACCTGATGAAGATCCTGAC	[36]
ACTIN-R	GCACAGCTTTTCCTTGATGTCGC	

### *Cx. tarsalis* immune gene expression in response to *Wolbachia*

To explore *Wolbachia* effects on mosquito immune gene expression, one- to four- day old adult female *Cx. tarsalis* were anesthetized with CO_2_ and injected as described above with *Wolbachia* (*w*AlbB) or Schneider's insect media as control. Mosquitoes were provided with 10% sucrose *ad libitum* and maintained at 27°C in a growth chamber. At 7 dpi, mosquitoes were blood fed on bovine blood via glass membrane feeder. At 2 dpf, five mosquitoes per treatment were harvested and RNA extracted using RNeasy mini kits (Qiagen). Extracted RNA was DNase treated (Ambion #AM1906) and converted to cDNA using Superscript III with random hexamers (Invitrogen #18080-51) according to the manufacturers' protocols. qPCRs were performed using the Rotor Gene Q (Qiagen) and SYBR Green qPCR kit (Qiagen) according to the manufacturer's protocol. Five target immune genes in the Toll and IMD innate immune pathways (REL1, REL2, cactus, defensin and diptericin) were selected, primers designed based on homologous genes in the *Anopheles gambiae*, *Aedes aegypti* and *Culex pipiens* genomes and normalized to host actin (Table 1). Gene expression was analyzed by calculating ratios of target to host gene and tested for significance using Mann-Whitney U test. All qPCRs were technically replicated twice.

## Results

### Fluorescence *in situ* hybridization (FISH)

**Figure 1:**
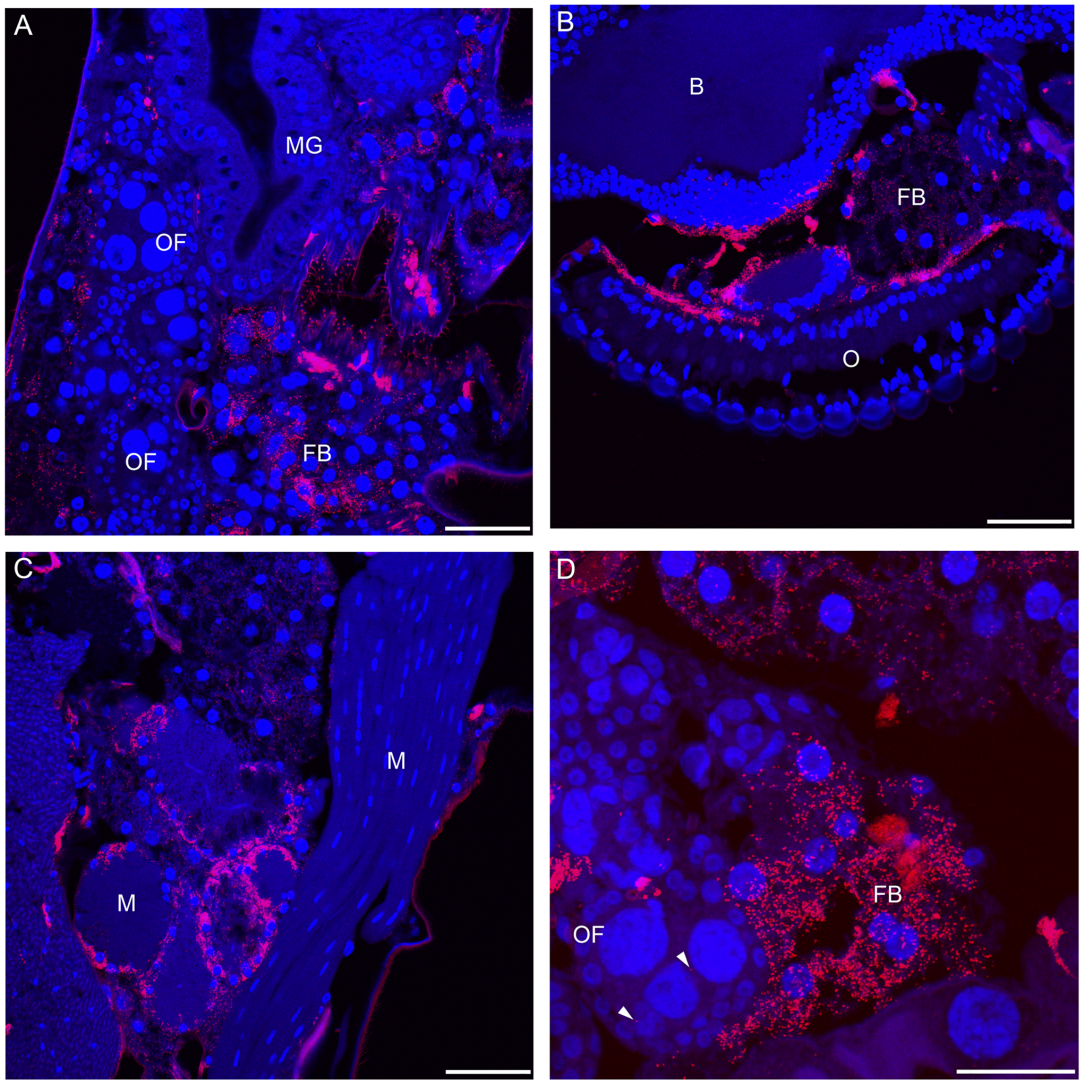
Fluorescence *in situ* hybridization of *Wolbachia* infection in *Cx. tarsalis* mosquitoes 12 days post injection. Confocal microscopy of sectioned mosquitoes shows *Wolbachia* infection in diverse tissues after adult microinjection. A. *Wolbachia* localized in the abdomen of *Cx. tarsalis*. B. *Wolbachia* infection disseminated to the head and nervous tissue. C. *Wolbachia* is present in the muscular tissue of the mosquito. D. *Wolbachia* infection within and surrounding the ovarian follicles. Arrowheads denote infection within the ovarian follicle. The scale bar represents 50 um. OF; ovarian follicle, MG; midgut, FB; fat body, M; muscle, B; brain, O; omnitidia. Red = *Wolbachia*; Blue = mosquito DNA.

Using fluorescence *in situ* hybridization, we observed that *w*AlbB establishes an infection in both somatic and germline tissue in *Cx. tarsalis* 12 days post injection. *Wolbachia* disseminated to various tissues including the head, proboscis, thoracic flight muscles, fat body and ovarian follicles (Figure 1). *Cx. tarsalis* appeared heavily infected, suggesting that adult microinjection is an effective method to experimentally infect this mosquito species.

### Vector competence for WNV

**Figure 2:**
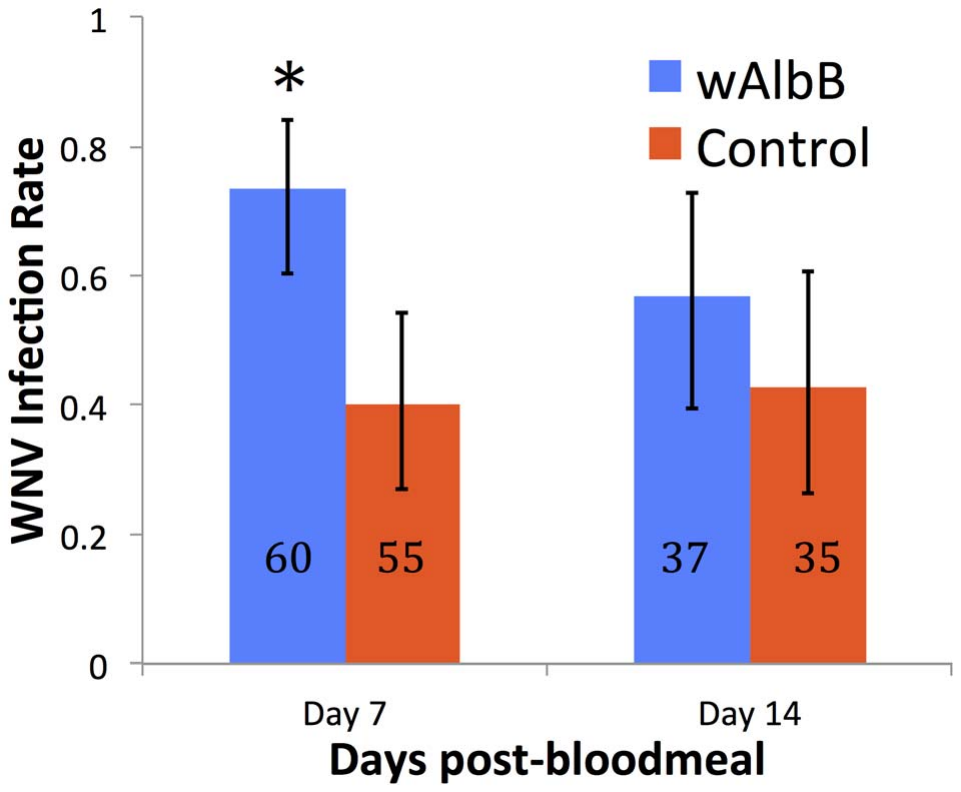
Effect of *w*AlbB infection status on WNV probability of infection in *Cx. tarsalis*. *w*AlbB infection significantly increases WNV infection 7 days post-bloodmeal. Asterisk denotes statistical significance (P = 0.04). N denotes sample size. Error bars represent 95% binomial confidence intervals.

We evaluated the vector competence of *Wolbachia*-infected and uninfected *Cx. tarsalis* for WNV in mosquito bodies, legs and salivary secretions to determine infection, dissemination and transmission rates, respectively. Replicate results were similar, and results from pooled replicates or analysis of individual replicates were identical, so the pooled analysis is presented for clarity; results from individual replicates are available as Table S1. *w*AlbB-infected *Cx. tarsalis* displayed significantly higher WNV infection rates 7 days post-feeding (dpf) (P = 0.04). A similar but non-significant trend was observed 14 dpf (Figure 2). If mosquitoes were infected, virus dissemination and transmission rates did not differ statistically (Table S1).

### Quantitative real-time PCR (qPCR) of *Wolbachia* density

**Figure 3:**
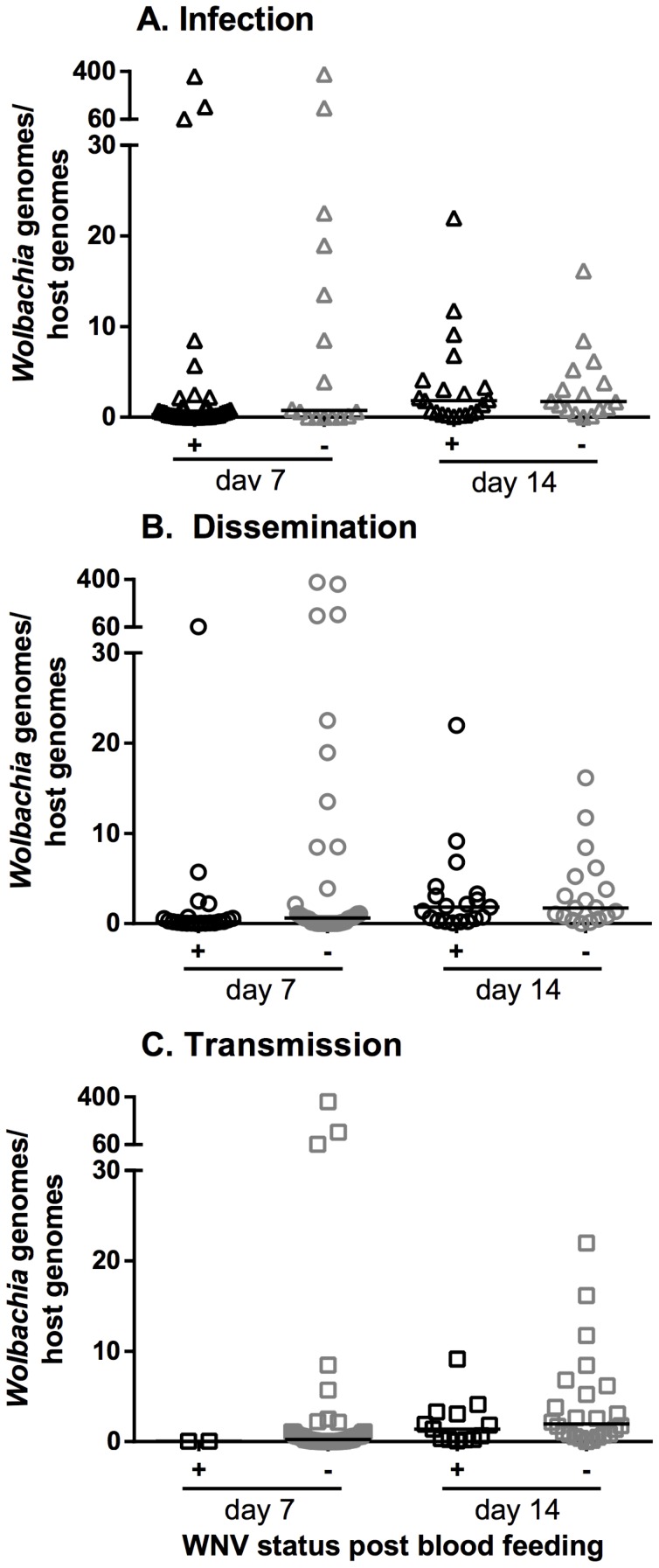
Comparison of WNV infection status and *Wolbachia* titers in *Cx. tarsalis*. *Wolbachia* titers in mosquito bodies were compared between WNV positive (black symbols) or negative (gray symbols) bodies, legs and salivary secretions. (A) Infection, (B) Dissemination, and (C) Transmission.

To determine if there was a *Wolbachia* density effect on WNV phenotype, qPCR was used to compare *Wolbachia* titers in mosquitoes either positive or negative WNV. *Wolbachia* titers in WNV-infected versus uninfected *Cx. tarsalis* did not differ statistically; similarly, no significant titer differences were found in individuals that disseminated versus non-disseminated or transmitted vs. non-transmitted (Figure 3).

### *Cx. tarsalis* immune gene expression in response to *Wolbachia*

**Figure 4:**
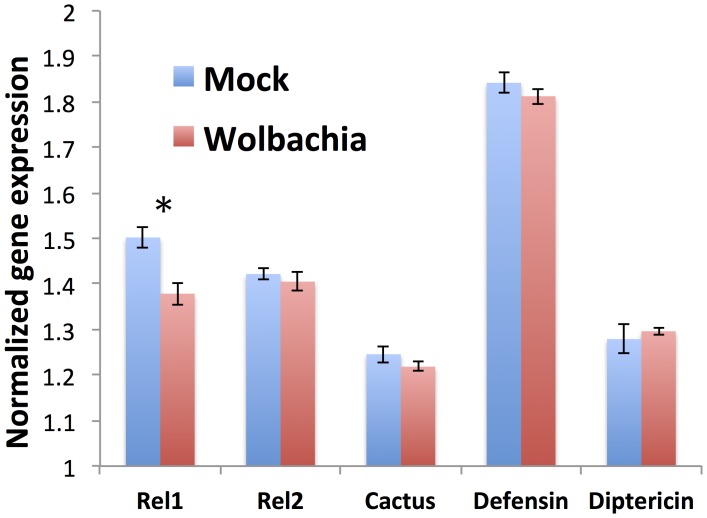
*Cx. tarsalis* immune gene expression in response to *Wolbachia* infection. Expression for each gene was normalized to the S7 gene. *Cx. tarsalis* females were injected with *Wolbachia* or media as control, blood fed 7 days post-injection and harvested 2 dpf to assess expression of five key immune genes. N = 5 per gene. REL1 expression in *Wolbachia* injected mosquitoes is significantly higher compared to control (one-tailed P = 0.008; asterisk). Error bars represent standard errors.

To elucidate the mechanism behind *Wolbachia* mediated WNV infection enhancement in *Cx. tarsalis*, we evaluated mosquito immune gene expression in response to *Wolbachia* using qPCR. Unlike other systems [38]–[40], *Wolbachia* did not induce a significant immune response in *Cx. tarsalis* females compared to the control. In contrast, REL1 (the NF kappa B activator of the antiviral Toll pathway) was significantly reduced in *Wolbachia*-infected mosquitoes compared to controls (one-tailed P = 0.008) (Figure 4).

## Discussion

### Caveats of this study

It should be noted that these experiments were performed with mosquitoes transiently infected in the somatic tissues with *Wolbachia*, rather than a stable maternally inherited infection. It remains to be seen whether a stable *w*AlbB infection will enhance WNV in a similar way. *Wolbachia* density in mosquito somatic tissues (as opposed to germline) was found to explain differences in virus infection in *Aedes* mosquitoes [41]. Thus, it seems likely that if stable infection in *Cx. tarsalis* has a similar somatic tissue distribution to a transient infection it may induce a similar virus enhancement phenotype. However, this must be tested empirically. It is also unknown whether virus enhancement is limited to WNV or occurs more broadly. Finally, we tested a single *Wolbachia* strain, and it is unknown whether virus enhancement is specific to *w*AlbB or occurs with diverse *Wolbachia* strains.

Previous studies have shown that pathogen suppression by *Wolbachia* has the potential to be a novel method for controlling vector-borne diseases [4], [42]–[44]. Not all mosquito species are naturally infected with *Wolbachia*, but non-infected species may support infection once introduced and these novel infections often effectively inhibit various pathogens [5], [45]. Our experiments indicate that following adult microinjection, *Wolbachia* is capable of establishing both somatic and germline infection in *Cx. tarsalis* but does not inhibit WNV infection, dissemination or transmission. In contrast with other studies showing pathogen inhibition by *Wolbachia*, our data suggest that *Wolbachia* may in fact increase WNV infection rates in *Cx. tarsalis*, particularly at early time points. Increased early infection has the potential to shorten the extrinsic incubation period of the pathogen, which can dramatically increase the reproductive rate of the virus [19]. It has become increasingly clear that *Wolbachia* does not always suppress pathogens in insects [46]. For example, the cereal crop pest *Spodoptera exempta* is more susceptible to nucleopolydrovirus mortality in the presence of *Wolbachia* [47]. In the mosquitoes *An. gambiae An. stephensi, Ae. fluviatilis and Cx. pipiens*, *Wolbachia* enhances *Plasmodium berghei, P. yoelii, P. gallinaceum and P. relictum*, respectively [17]–[20]. Enhancement may be dependent on the host-*Wolbachia* strain-pathogen system of interest, as *Wolbachia* strains that block one pathogen yet enhance another have been documented [9], [17]. *Wolbachia*-mediated pathogen enhancement may be a common yet often ignored phenomenon, which merits attention when designing *Wolbachia*-based strategies for disease control [46].

Intracellular infection with bacteria may alter the cellular environment in multiple ways, including bacterial manipulation to avoid host immune defenses [48]. Though the exact *Wolbachia*-mediated inhibition mechanism is unknown, studies have suggested that *Wolbachia* indirectly modulates mosquito immunity [40], [49]. *Wolbachia* can activate the Toll pathway, stimulating a cascade of events that have been correlated with inhibition of dengue and *Plasmodium* in mosquitoes [39], [50], [51]. In contrast, in *Cx. tarsalis*, *w*AlbB infection significantly downregulated REL1 (the activator of the Toll pathway), suggesting that in this system *Wolbachia* may down regulate antiviral Toll-based immunity leading to increased virus infection. However, while statistically significant, this decrease in REL1 expression was modest, and further study is required to determine the precise mechanism of *Wolbachia*-based WNV enhancement in this system.

To our knowledge this is first study showing *Wolbachia* can potentially enhance a vector-borne pathogen that causes human disease. Our results, combined with other *Wolbachia* enhancement studies [17]–[20], [46]–[47], suggest that field deployment of *Wolbachia*-infected mosquitoes should proceed with caution. *Wolbachia* effects on all potential pathogens in the study area should be determined. Additionally, several studies have shown that *Wolbachia* is capable of horizontal transfer to other insect species which could have unforeseen effects on non-target insects [52]–[54]. A lack of understanding of *Wolbachia*-pathogen-mosquito interactions could impact efficacy of disease control programs. *Cx. tarsalis* is a competent vector for many human pathogens, and further studies that assess alternative *Wolbachia* strains and viruses in *Cx. tarsalis* may elucidate the importance of host background on pathogen interference phenotypes in this medically important mosquito species.

## Supporting Information

Figure S1 **FISH controls. **Red: *Wolbachia* **, Blue: mosquito DNA, Green: background fluorescence. Top row: positive (** ***w*** **AlbB) control.**
(PDF)

Table S1 **Results from individual vector competence replicates.**
(PDF)
