# Supplementary figures and images for: Wolbachia Enhances West Nile Virus (WNV) Infection in the Mosquito Culex tarsalis
Source: PLoS Negl Trop Dis. 2014 Jul 10;8(7):e2965. doi: 10.1371/journal.pntd.0002965 (PMC4091933; doi:10.1371/journal.pntd.0002965)

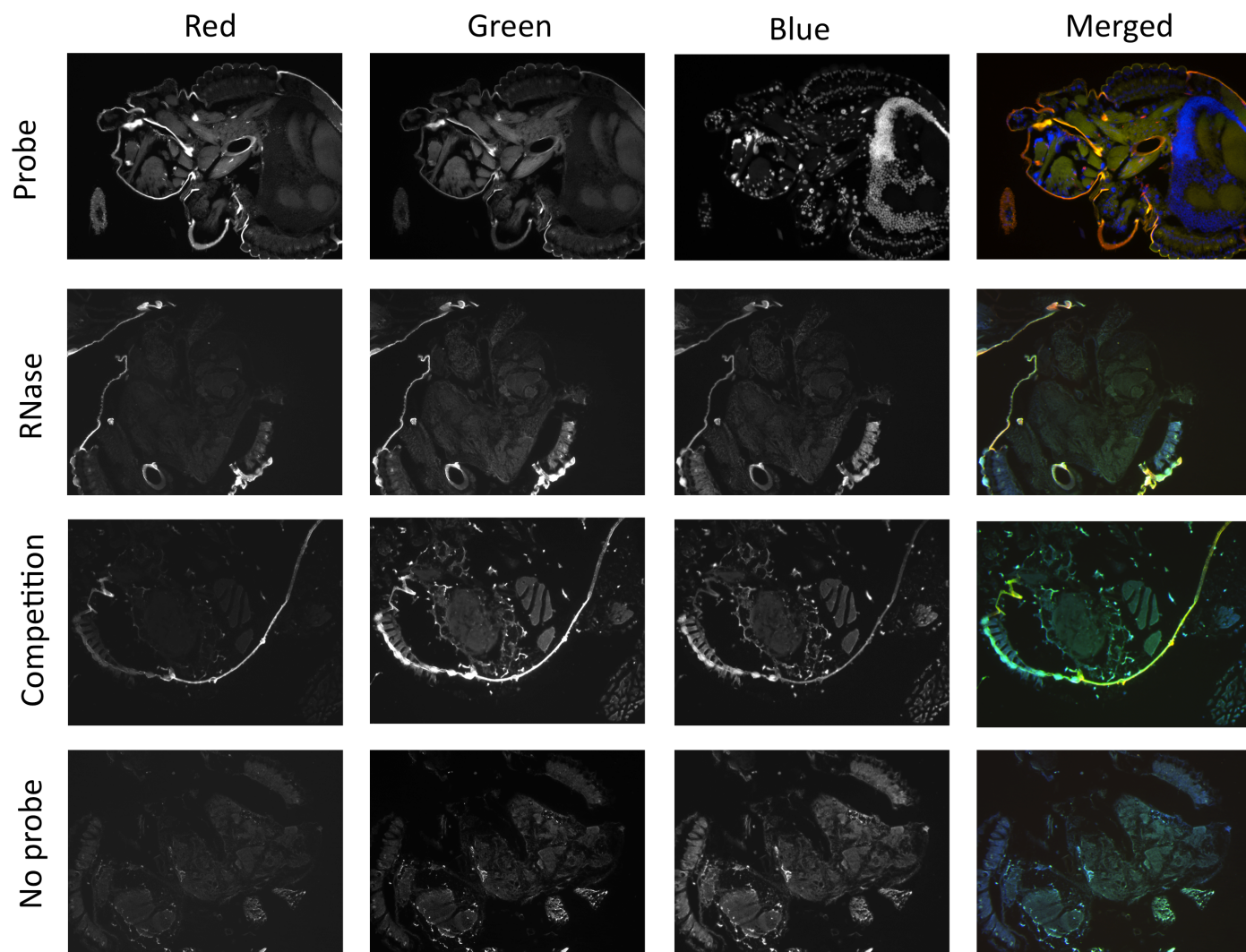

Supplement: Figure S1 — FISH controls. Red: Wolbachia , Blue: mosquito DNA, Green: background fluorescence. Top row: positive ( w AlbB) control. (PDF) [file pntd.0002965.s001.pdf]
