# Supplementary material for: Wolbachia Enhances West Nile Virus (WNV) Infection in the Mosquito Culex tarsalis
Source: PLoS Negl Trop Dis. 2014 Jul 10;8(7):e2965. doi: 10.1371/journal.pntd.0002965 (PMC4091933; doi:10.1371/journal.pntd.0002965)
Supplement: Table S1 — Results from individual vector competence replicates. (PDF) [file pntd.0002965.s002.pdf]

Supporting Table 1. Vector competence of *Culex tarsalis* following a WNV infectious blood meal. Parentheses denote sample sizes.

Treatment	Day post feeding	Rep 1	Rep 2
	**Infected**		
Wolbachia	7	73 (30)	73 (30)
Control		43 (30)	36 (25)
p value[b]		***0.035***	***0.007***
Wolbachia	14	50 (30)	86 (7)
Control		47 (30)	20 (5)
p value[b]		1.0	0.072
	**Disseminated**		
Wolbachia	7	55	40
Control		38	56
p value[b]		0.49	0.69
Wolbachia	14	93	83
Control		86	100
p value[b]		0.598	1.0
	**Transmitted**		
Wolbachia	7	5	0
Control		0	11
p value[b]		1.0	0.29
Wolbachia	14	60	67
Control		36	100
p value[b]		0.27	1.0

[a] Calculated based on number of infected mosquitoes
[b] Fisher's exact test
Italicized and bold values denote statistical significance.
